# Emerging professional practices focusing on reducing inequity in speech-language therapy and audiology: a scoping review

**DOI:** 10.1186/s12939-022-01815-0

**Published:** 2023-03-10

**Authors:** Kristen Abrahams, Rizwana Mallick, Ameer S-J Hohlfeld, Thiani Pillay, Tamzyn Suliaman, Harsha Kathard

**Affiliations:** 1 Division of Communication Sciences and Disorders, Department of Health and Rehabilitation Sciences, University of Cape Town, South Africa, F45 Old Main Building, Groote Schuur Hospital, Observatory, Cape Town, South Africa; 2 South African Medical Research Council, Cochrane South Africa, Francie Van Zijl Drive, Parowvallei, Tygerberg, PO Box 19070, Cape Town, 7505 South Africa; 3 Discipline of Speech Language Pathology, University of Kwa-Zulu Natal, College of Health Sciences, KwaZulu Natal, University Road, Westville, Private Bag X 54001, Durban, 4000 South Africa; 4 University of Cape Town, UCT Libraries, Chancellor Oppenheimer Library, North Lane, Rondebosch, Cape Town, 7701 South Africa; 5 Inclusive Practices Africa Research Unit, University of Cape Town, Private Bag X3, Rondebosch, Cape Town, 7701 South Africa

**Keywords:** Health equity, Decoloniality, Communication, Global South

## Abstract

**Background:**

For the professions of audiology and speech-language therapy (A/SLT), there continues be a dire need for more equitable services. Therefore there is a need to develop emerging practices which have a specific focus on equity as a driving force in shifting practices. This scoping review aimed to synthesise the characteristics of emerging practices in A/SLT clinical practice in relation to equity with an emphasis on communication professions.

**Methods:**

This scoping review followed the Joanna Briggs Institute guidelines and aimed to map the emerging practices in A/SLT to identify the ways in which the professions are developing equitable practices. Papers were included if they addressed equity, focused on clinical practice and were situated within A/SLT literature. There were no time or language restrictions. The review included all sources of evidence across PubMed, Scopus, EbscoHost, The Cochrane Library and Dissertation Abstracts International, Education Resource Information Centre from their inception. The review uses PRISMA Extension for scoping reviews and PRISMA-Equity Extension reporting guidelines.

**Results:**

The 20 included studies ranged from 1997–2020, spanning over 20 years. There were a variety of papers including empirical studies, commentaries, reviews and research. The results demonstrated that the professions were increasingly considering addressing equity through their practice. However, there was a prominent focus around culturally and linguistically diverse populations, with limited engagement around other intersections of marginalisation. The results also showed that while the majority of contributions to theorising equity are from the Global North with a small cluster from the Global South offering critical contributions considering social categories such as race and class. Collectively the contributions from the Global South remain a very small minority of the professional discourse which have a focus on equity.

**Conclusion:**

Over the last eight years, the A/SLT professions are increasingly developing emerging practices to advance equity by engaging with marginalised communities. However, the professions have a long way to go to achieve equitable practice. The decolonial lens acknowledges the impact and influence of colonisation and coloniality in shaping inequity. Using this lens, we argue for the need to consider communication as a key aspect of health necessary to achieve health equity.

**Supplementary Information:**

The online version contains supplementary material available at 10.1186/s12939-022-01815-0.

## Introduction

It is well documented that health inequities are a consequence of systemic social injustices which are intrinsically linked to the longstanding histories of social, political and structural systems [1]. The impact of colonisation and the pervasive effects of coloniality have shaped and directly contributed to the critical health inequities evident in the struggles of post-colonies [2]. While much has been written about health disparities from a biomedical perspective [1, 3, 4], this paper specifically focuses on communication as an essential part of health.

While health disparities have been the focus of the professions (i.e. understanding the social injustices and social disadvantage which contribute to health differences and outcomes), this paper shifts focus to health equity – the systemic processes and actions which lead to health equity [5]. In arguing for a shift from a health disparity focus (problem identification focus) to a health equity focus (solution focus), Srinivasan and Williams [6] argued that health equity aspires to the highest level of health for all people. This requires a focused strategy of change which addresses avoidable inequalities created through historical and contemporary injustices at a population level. It is this understanding that we explore how inequity has been conceptualised in A/SLT.

### Understanding inequity in audiology and speech-language therapy

Communication is joint human meaning-making essential for socialisation, learning, and earning through multiple modalities including written, verbal, manual, and digital. Traditionally communication challenges have been linked to specific communication disorders or disabilities [7] e.g., hearing impairment, language learning disorders, stuttering. Additionally, a large number of people in contexts of social disadvantage (e.g., poverty, migration, displacement, gender diversity, race) struggle with developing communication required for inclusion as they struggle with opportunities to acquire complex language in particular the language of dominance e.g., English [8]. While there are no estimates of the prevalence of people with communication challenges, the World Report on Disability [9] estimates 15% of the world’s population are persons with disabilities. Cieza, Causey [10] further estimated 2/3 people will require rehabilitation services at some point in their lives. Persons who have communication challenges also struggle to access health services related to their conditions as well as general health care in a health system – that has typically been designed for able-bodied people with interventions being biomedical [11]. The majority of the people in need of service provision are from the Global South /Majority world [12].

Within the Western health system, persons with communication challenges rely on speech-language-hearing professions (Audiology and Speech-language Therapy [A/SLT]) for services. The A/SLT professions were established in the Global North (and minimally in the Global South) within a colonially-inspired service model which has been largely Western, middle class, individualised, medicalised, monolingual English and mainly for people who can afford services [13]. The professional workforce is also mainly located in the Global North. The therapist-patient ratio varies in the Global North from approximately 1:2500 to 1:4700 [14].

In the Global South by comparison, the Majority world has little or poorly established services [15, 16]. South Africa is perhaps an exception as the profession was established approximately 80 years ago during the apartheid era and mainly serviced privileged White communities [15, 17]. Perhaps the most limiting aspect of the service model in the Global North and South is its unwavering reliance on an individual model compared to a population-based approach [18].

As a consequence, a large part of the world’s population remains underserved. Furthermore, even where services are established, such as in South Africa, part of the population remains underserved or inappropriately/inadequately served. For example, populations of people of language and cultural backgrounds, including black, gender diverse communities remain underserved. Continuing to practice using this colonised model of practice is untenable as it contributes to perpetuating inequities across the world [17].

Given the dire need for equitable service delivery, the professions of A/SLT are challenged to develop emerging practices i.e., to break away from the traditional ways of practicing and to use equity as a driver for reshaping practices. In the context of this scoping review, emerging practices are understood as those practices which are developing, changing and adapting from the traditional individual, institutionalised model of practice. While emerging practices are borne out of traditional practice, they begin to shift away from the traditional model through innovation and creativity [19].

Against this backdrop – we question: how are the professions of A/SLT developing emerging practices which make advances to health equity? This scoping review seeks to understand how the professions are engaging with the issue of equity and what the innovations and drivers are for changing practice. Through this analysis, we aim to generate a conceptual framework for the emerging practices. Given that health equity is influenced by deeply entrenched colonial systems, values and processes, we use a decolonial lens to offer a deeper level of analysis. The decolonial lens is useful because it brings into consciousness and makes visible the values, norms, customs imposed by the colonisers [20]. The decolonial lens also provides the impetus to creatively disrupt and delink from inequitable practices which have become naturalised and deeply entrenched.

## Methods

A study protocol [21] was developed following the methodological procedures for scoping reviews as proposed by the Joanna Briggs Institute [22] and is reported in accordance with the Preferred Reporting Items for Systematic Reviews and Meta-Analysis Extension for scoping reviews (PRISMA-Scr) statement [23] and the PRISMA-Equity Extension [24].

### Aim/Objectives

The following research aims/objectives were developed:

- 1. To synthesise the characteristics of emerging practices in A/SLT clinical practice in relation to equity
-
  a To synthesise the ways in which equity is defined in the professions.
  b To identify and describe innovations in practices.
  c To understand and describe the drivers for change in clinical practice.
  d Develop a conceptual understanding of emerging practice based on the literature.

### Identifying relevant studies

#### Eligibility criteria

Studies across all sources of evidence including primary studies, peer reviewed research studies, opinion pieces, book chapters, empirical studies, conceptual papers, and grey literature were included in order to understand the breadth of literature. Papers needed to show a clear focus on clinical practice. For this scoping review, clinical practice was defined as activities performed by a professional and the resources used to achieve such practice activities. During the screening process, this definition of clinical practice was extended to praxis which acknowledges the continuous interplay between thought and action. As such we included both conceptual and practice-based literature in the scoping review. Papers were included if they had a specific focus on human communication and drew links to A/SLT literature. There were no language limitations and the authors translated abstracts of papers within their capacity and funding available. There was no time restriction on the date of publication.

In addition, papers needed to address equity. For the study, “equity in health can be defined as the absence of disparities in health (and in its key social determinants) that are systematically associated with social advantage/disadvantage” [3] (pg. 256). The scoping review specifically considered practices focusing on marginalised communities as defined through the lens of equity. In addition, we understood that inequity spans across all geographic locations and as such we focused on Global South context. We understood the Global South as countries that experience exploitation, marginalisation, and oppression. As the Global South is defined by geo-political boundaries, we did not limit the search to geographic location. The links to the Global South were determined during screening and analysis process based on the populations focus of the papers (e.g. migrant, Indigenous populations).

Through the screening process, it became clear that we needed to reframe the concept of equity. The complexity of the concept needed clarification and therefore we drew on intersectionality and marginalisation as key framings. Using intersectionality, we understood that social categories (race, gender, class, sexuality etc.) interact to create the context for inequity. We placed particular emphasis on the importance of the interaction i.e., multiple social categories may interact and result in marginalisation [25]. We further realised the importance of defining the concept of marginalisation as a core concept in understanding equity. For the study, marginalisation was understood as “the peripheralisation of individuals and groups from a dominant, central majority” [26] (pg. 46). Within the professions, those who benefit the most from A/SLT services are generally white, middle-class populations who speak a dominant language [13]. In this context, marginalisation is understood as those populations who fall outside/on the fringes of those social categories (e.g., poor, black, indigenous populations).

Papers were excluded when there was no clear intention for equity, clinical practice, and communication. While we acknowledge that for the A/SLT professions swallowing and balance form a part of our scope of practice, papers with a sole focus on these areas were outside of the scope of the review as the focus was on communication.

#### Search strategy

A general search was used to inform the following: (1) to identify where any similar scoping reviews had already been conducted and when it was conducted; (2) to refine the inclusion and exclusion criteria; and (3) to determine the viability of the topic.

A three-step search strategy was used [22]: (1) librarian (TS) was consulted to assist with refining the research question, identification of databases and the development of an initial search strategy; (2) an initial limited search of two online databases, namely Scopus and EbscoHost, was conducted. This search was used to identify relevant keywords through reviewing title, abstracts and index terms; and (3) TS conducted the second search using the keywords identified in the initial search across all of the databases to identify relevant paper for consideration in the review in March 2021. Throughout this process, the refining of the search strategy was iterative. Additional search terms were incorporated into the search strategy throughout the initial searches until the final search strategy was developed.

The following databases were included in the search: *PubMed* (https://pubmed.ncbi.nlm.nih.gov/)*, Scopus* (https://www.scopus.com/)*, EbscoHost* [https://search.ebscohost.com/Login.aspx, including Academic Search Premier, Africa-wide Information, Cumulative Index to Nursing and Allied Health, Education Resources Information Center, Health Source (consumer edition)], *The Cochrane Library* (https://www.cochranelibrary.com/ including Cochrane Database of Systematic Reviews, Cochrane Central Register of Controlled Trials and Cochrane Methodology Register) and *Dissertation Abstracts International, Education Resource Information Centre* (http://ezproxy.uct.ac.za/login?url=https://search.ebscohost.com/login.asp?profile=ehost&defaultdb=eric)*.* All databases were search from their inception and no filters were used. The reference lists of the included papers were reviewed to identify any additional papers. We did not specifically search for grey literature but did not exclude grey literature in the development of the search strategy or the final search results. See Additional file 1 for the final search strategy.

### Selecting studies for inclusion

#### Screening of records

The reviewers used the protocol developed to guide the selection process for the sources of evidence. Endnote 20 was used to manage the results of the search. In addition, Rayyan was used to assist with the screening process [27]. In order to increase the consistency amongst the reviewers, the study protocol was piloted on a random sample of 25 titles/abstracts. Two reviewers (KA, RM) independently screened the title/abstracts using the eligibility criteria and definitions. No changes were made to the protocol following the piloting.

Using Rayyan [27], KA, and RM independently screened paper titles and abstracts using the inclusion and exclusion criteria. KA and RM resolved any discrepancies in study selection through discussion and joint decision making. If there was uncertainty about the title/abstracts, papers were included for full text review. Following which, KA and RM screened full text papers to determine if they met the inclusion and exclusion criteria. The reviewers consulted each other to determine if there were any disagreements in records selected for inclusion. Where no agreement could be reached, a third reviewer (HK) was consulted to assist with reaching a consensus.

The data extraction and data analysis occurred concurrently. In order to refine the data analysis template developed, the reviewers (KA, RM, HK, TP) independently reviewed one of the included papers to identify key themes emerging in line with the aims and objectives (See Additional file 2 for the final data extraction tool). The data analysis specifically considered the lens in which the papers addressed equity, the drivers for change (be it health equity or disparity), the type of practice described, and documented the innovations (i.e. the shifts from traditional practice). For the qualitative analysis, thematic analysis was used [28]. Two reviewers independently documented key themes emerging from the papers for objectives one, two and three. In this process, we also considered the different levels of analysis i.e., interpretation and critical interpretation using decoloniality specifically the colonial matrix of power [29] and the Ecology of Human Performance framework [30] as theoretical framings [31]. In order to extract the key themes across papers, the reviewers collectively discussed each paper and noted similarities and differences. Based on these discussions the key themes across the levels of analysis were finalised. These discussions formed the basis for developing a conceptual understanding of emerging practices as outlined in Objective 4.

## Results

**Fig. 1:**
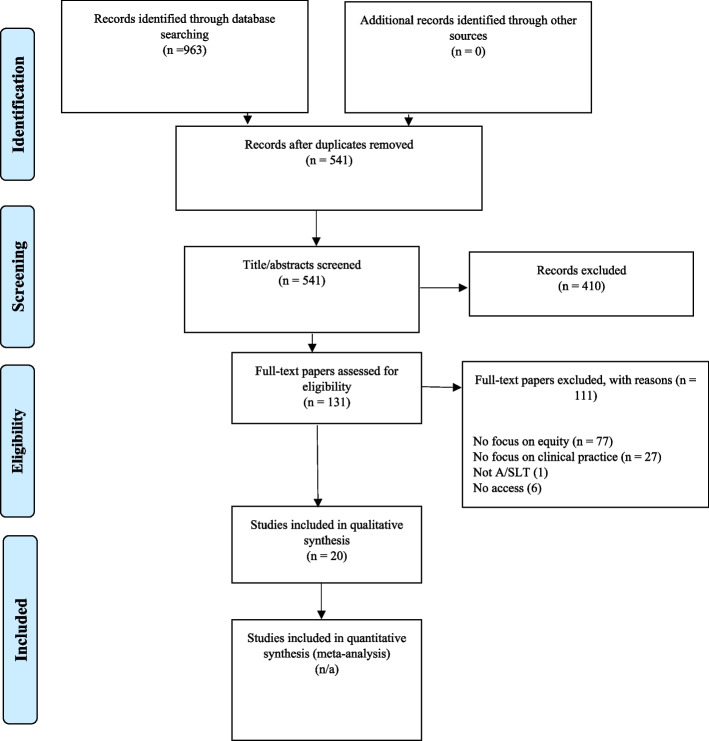
Review flow diagram for study selection [23]

A total of 963 records were identified in the search of the databases. There were 541 records which were included in the title and abstract screening. Almost a quarter (*n* = 131/541; 24%) of the records were eligible for inclusion for the full text screening. Of the 131, we were able to access 125 full texts and as such 6 papers were excluded due to access. Figure 1 documented the 20 papers which were included in the scoping review for qualitative analysis (See Additional file 3 for list of included papers and Additional file 4 for reasons for exclusion).

### Key characteristics of included studies

Characteristics of the included papers (*n* = 20) were extracted based on key descriptors. Table 1 summarises the basic information including author, type of publication, geographical context, Global North/South and the population focus. This allowed us to understand the dispersion of papers across a range of descriptions and gave us insight into the existing trends in the research on the topic.

**Table 1:** Characteristics of included studies presented in chronological order (*n* = 20)

Date	Author	Type of publication	Geographical Context	Global North/South	Population focus (intersections)
1997	Pillay [32]	Empirical study	South Africa	South	Structural disadvantage (race, language)
2010	Fuller [33]	Research	United Kingdom	South	Disadvantage children [socio-economic (SE)]
2013	Cheng [39]	Commentary	USA	South	China/Taiwan (language, culture, ability)
2013	Davidson [40]	Commentary	Australia	South	Aboriginal/Torres Strait Islanders (language, culture, SE, ability)
2013	Kathard [8]	Commentary	South Africa	South	Structural disadvantage (race, SE, language, ability)
2013	Westby [41]	Commentary	USA	South	Unspecified (language, culture, SE, ability)
2014	Hyter [42]	Not reported	USA	South	Unspecified (race, language, culture, SE, ability)
2014	Penn [47]	Not reported	South Africa	South	Structural disadvantage (SE, language, ability)
2016	Grech [43]	Not reported	Malta	South	Migrant population (language, culture, SE, citizenship)
2017	Brewer [44]	Commentary	New Zealand	South	Maori (language, culture, SE)
2017	Dressel [45]	Short Report	USA	South	Malawi (SE, ability)
2017	Penn [48]	Commentary	South Africa	South	Aboriginal people with communication disability (PWCD) (language, culture, SE, ability)
2018	Carroll [50]	Commentary	Ireland	North	PWCD (ability)
2018	Hopf [38]	Commentary	Australia	South	Fiji PWCD (language, culture, SE, ability)
2018	Pascoe [46]	Review	South Africa	South	Culturally and linguistically diverse populations (language, culture)
2018	Pillay [18]	Not reported	South Africa	South	Structural disadvantage (race, SE, language)
2019	Suen [51]	Review	USA	Unspecified	Hearing health (pathology)
2019	Abrahams [17]	Not reported	South Africa	South	Structural disadvantage (race, SE, language, culture)
2020	Bondurant [52]	Research	USA	Unspecified	Unspecified
2020	Merritt [49]	Commentary	USA	North	Gender diverse (gender, ability)

^*^USA United States of America, *SE* socio-economic, *PWCD* people with communication disorders

Included papers were published between one and twenty-four years ago. However, the gap between the two oldest papers [32, 33] is more than thirteen years. While the first paper was published in South Africa in 1997, the majority (*n* = 18/20; 90%) of the papers were published in the last eight years. These results potentially show a growing recent interest in the field around equity within A/SLT research.

Geographically, the results were dispersed across several countries. Seven of the papers (*n* = 7/20; 35%) were published from South Africa and the United States respectively. The rest of the papers were dispersed amongst countries including Australia (*n* = 2/20; 10%), New Zealand (*n* = 1/20; 5%), Malta (*n* = 1/20; 5%), Ireland (*n* = 1/20; 5%) and the United Kingdom (*n* = 1/20; 5%). Over half of the included papers (*n* = 13/20; 65%) were produced in deemed high-income countries [34]. While seven (*n* = 7/20; 35%) papers were produced in low-middle income countries (all from South Africa).

The diversity of the definitions of equity used in the included papers were often interlinked with the populations they chose to target within their interventions. These populations were often determined as exploited, marginalised or oppressed population groups which are often described as the ‘Global South’ [35]. The Global South accounted for sixteen (*n* = 16/20; 80%) of the included papers.

In terms of the population focus, we focused on understanding the ways in which populations were marginalised. The majority of the papers considered the intersections of language (*n* = 14/20, 70%), socioeconomic status (*n* = 14/20, 70%), ability/disability (*n* = 12/20, 60%) and culture (*n* = 10/20, 50%) with emerging intersections including race (*n* = 5/20, 25%), citizenship (*n* = 1/20, 5%) and gender (*n* = 1/20, 5%). These results are in alignment with the current dominant focus of the literature in A/SLT, particularly around serving those with impairments and culturally and linguistically diverse populations [36].

### Results linked to aims and objectives

In Table 2 we extracted descriptive data related to the aims and objectives of this review, specifically around equity, drivers for change and practice innovations.

**Table 2:** Characteristics of included papers documented in chronological order (*n* = 20)

	**Year**	**Author**	**Equity**	**Drivers for change**	**Practice descriptor**	**Practice Innovation**
1	1997	Pillay [32]	Underserved populations	Health equity	Conceptual framework	Epistemological shift
2	2010	Fuller [33]	Underserved populations (vulnerable)	Health disparity	Clinical practice	Inclusivity
3	2013	Cheng [39]	Underserved populations	Health equity	Practice guidelines	Contextualising practice – marco level
4	2013	Davidson [40]	Human rights	Health disparity	Interprofessional Practice	Breaking down professional boundaries
5	2013	Kathard [8]	Underserved populations	Health equity	Conceptual framework	Epistemological shift
6	2013	Westby [41]	Underserved populations	Health equity	Conceptual framework	Epistemological shift
7	2014	Hyter [42]	Underserved populations	Health disparity	Conceptual framework	Epistemological shift
8	2014	Penn [47]	Public health	Health equity	Practice guidelines	Contextualising practice
9	2016	Grech [43]	Human rights	Health disparity	Conceptual framework	Epistemological shift
10	2017	Brewer [44]	Underserved populations	Health disparity	Practice guidelines	Contextualising practice
11	2017	Dressel [45]	Underserved populations (vulnerable)	Health disparity	Interprofessional practice	Breaking down professional boundaries
12	2017	Penn [48]	Human rights/decolonial	Health equity	Practice guidelines	Contextualising practice
13	2018	Carroll [50]	Human rights	Health disparity	Research/Practice intersection	Community engagement
14	2018	Hopf [38]	Human rights	Health disparity	Research/Practice intersection	Community engagement
15	2018	Pascoe [46]	Human rights	Health disparity	Practice guidelines	Contextualising practice
16	2018	Pillay [18]	Decolonial	Health equity	Conceptual framework	Epistemological shift
17	2019	Suen [51]	Public health	Health disparity	Research/Practice intersection	Community engagement
18	2019	Abrahams [17]	Decolonial	Health equity	Conceptual framework	Epistemological shift
19	2020	Bondurant [52]	Underserved populations	Health disparity	Interprofessional Practice	Breaking down professional boundaries
20	2020	Merritt [49]	Underserved populations	Health disparity	Clinical practice	Inclusivity

#### Exploring equity in the professions

Equity was illuminated through several parameters which included the servicing of underserved populations (*n* = 10/20; 50%), human rights (*n* = 6/20; 30%), public health (*n* = 2/20; 10%) and decoloniality (*n* = 3/20; 15%). Through exploring marginalisation, human rights and decoloniality, the professions are beginning to explore the impact of social, cultural, political, and economic factors which create and sustain inequity. This movement shows a developing professional political consciousness in acknowledging the vast challenge of unserved/underserved/marginalised populations [8].

#### Drivers for change

The key drivers for change were determined based on how equity was addressed relative to health inequity or health disparity as per Braveman [5]. Eight (*n* = 8/20; 40%) papers made reference to inequity through the lens of health inequity, with the remainder of the papers (*n* = 12/20; 60%) referring to equity through the lens of health disparity. Therefore, it appears that there is greater focus on naming the disparities (i.e. problem) than focusing on addressing inequity (solution).

#### Understanding practice innovations

The types of emerging practices ranged from the development of conceptual frameworks (*n* = 7, 35%) and practice guidelines (*n* = 5, 25%) to exploring interprofessional practices across professional and educational settings (*n* = 3, 15%), the intersections between research and practice (i.e., the ways in which research can inform practice, *n* = 3, 15%) and clinical practices with underserved populations (*n* = 2, 10%). This range of practices indicate that while the professions are making gains in conceptual thinking on addressing inequity, the implementation of practices are forthcoming and are challenging because they require fundamental shifts in practice.

We further sought to understand the innovations in these practices by considering the shifts from the traditional model of practice. Out of the twenty papers, the majority of the papers advanced practice innovations around epistemological/ideological shifts in thinking (*n* = 7, 35%) and contextualising practices beyond the traditional framing e.g., macro level engagements, considerations around populations served (*n* = 5, 25%). The remainder of the papers considered professional collaborations (e.g. A/SLT, occupational therapist, physiotherapist, nurse) and the importance of breaking down professional boundaries (*n* = 3, 15%), community engagement (*n* = 3, 15%), and developing inclusive professional practices (*n* = 2, 10%). Innovations ranged from adaptations to practice to more substantial epistemological shifts, which argued for more radical shifts in practice.

## Discussion

### Synthesis of findings

The scoping review aimed to understand emerging practices that had a specific focus on reducing health inequity. The discussion draws together the different levels of analysis [31] presented in the results section and provides a critical interpretation of the findings using the colonial matrix of power to deepen our understanding of inequity in the professions as linked to coloniality [29].

The colonial matrix of power allowed us to understand the link between dominance and marginalisation within society and acknowledge the inextricable links between knowledge and power. The colonial matrix of power is deeply entangled with the production of knowledge in Africa specifically, and the Global South more broadly [37] and illuminates the forces which dominate knowledge production (as white, male, western, capitalist etc.) which masquerade as an objective, universal truth.

The colonial matrix of power also illuminates issues of identity due to the ways in which race underpins the Western construction of the world [37]. Similarly, within A/SLT profession, its foundations and knowledge are entangled with coloniality and the nine parameters of the matrix of power, which continue to be maintained [17]. Using this framing, we explore the key issues around dominance, intersectional identity and health equity as the emerging themes emanating from the review.

### The multiple voices on equity

The largest body of literature informing clinical practices on equity in A/SLT has emanated from the Global North as documented in Table 1. There may be a number of reasons which contribute to this dominance beyond the origins of the profession, including opportunities to do research, access to funding, and the interests of journals. Within this review, many of the papers explored how the Global North can contribute to the development of practices in Global South [38–45] through student clinical placements and research. Other papers in the dataset while positioned in the Global South, used policies and practice guidelines developed in the Global North to inform how practice is developed/conceptualised in the Global South [46–48]. In other words, while positioned in the Global South, the papers relied on Global North practice routines.

There was no literature emanating from the Global South outside of the South African context. The results speak to the potential power of the North in dictating how practices evolve. Evidence-based practice guidelines around equity are therefore being developed and dominated by Global North perceptions on the Global South. Ndlovu-Gatsheni [37] argues that “African people have continued to be major consumers of ideas generated in the West and tested on the African soil and on African minds” (pg. 53). Such dominance highlights how coloniality continues to manifest through the producers and production of knowledge. It is particularly important to explore ways in which we break from the dominance of Western thought which “consistently subordinate African [Global South] voices and cries for freedom” [37] (pg. 59).

The freedom of the Global South to think and develop practices which address marginalisation is particularly important. Marginalisation is not an apolitical process. It was largely created by colonisation and sustained through coloniality as many communities continue to suffer discrimination and oppression. It is particularly important to understand marginalisation through the lens of coloniality particularly in the Global South as there is danger of thinking about marginalisation as a neutral process. The tangible absence of the Global South in developing practices arises from the fact that there are few A/SLT practicing in the Global South, and where they are trying to change, the change is occurring through practice. It becomes imperative for the Global South to invest in developing new practices as there is a danger of replication where the dominant practice model is uncritically applied. However, we are hopeful that there is critical engagement between the Global North and South to advance equitable practices relevant to contexts.

### Health equity and health disparity

Within this review, there was a strong focus on health disparity compared to health equity. Health disparity references the differences in health amongst socially and economically disadvantaged people. An acknowledgement of the systemic challenges to accessing health care based on socio-economic status, disability, citizenship, culture, language, gender, race and geographic location (among other characteristics) associated with discrimination and exclusion [5] were prefaced in the dataset. This leads us to understand that the current literature is able to identify the underserved populations along the intersections of matrix of power (problem identification approach) in an attempt to address this service inequity. In our emerging practices, the dominant trend is around naming the problem with a prominent focus on culturally and linguistically diverse populations [38, 39, 41, 46].

There are however shifts toward a focus on health equity [8, 18, 19, 32, 39, 41, 47, 48], where authors are considering how we begin to address these health disparities to achieve the highest health possible for all regardless of social and economic differences (i.e., solution-orientated approach). Srinivasan and Williams [6] emphasised the shift from health disparity to health equity requires shifting the research agenda to consider population-based interventions. This shows the need in the professions to consider how we begin to think about and develop practices which are population-based, and inclusive, which work toward achieving health equity.

### Expanding intersectional identity in health equity

The dominant focus in the literature addressing equity has been around the intersections of culture and language and the majority of the papers have placed particular emphasis on working with culturally and linguistically diverse populations. Emerging trends which consider other social categories show that the profession is beginning to explore other intersections of identity beyond language and culture to acknowledge the importance of gender [49], race [32], and citizenship [43] as key areas of marginalisation. This is an important shifting from the dominant narrative of our professions.

Interestingly, when exploring these identities, papers largely positioned marginalisation from the perspective of the patient/community/population being served without reflecting on the value system of the profession itself. In previous works, we explored the profession as a project of coloniality [17] acknowledging how our practices are embedded in white, middle class, western norms and values. While it is useful to shift that dominant narrative by exploring issues of gender, race and other social categories, it is equally important to acknowledge that our work in the profession is not neutral and is informed and maintained by a white, monolingual, monocultural narrative. By acknowledging and understanding the positionality of the profession, we can create a context for understanding how our practices continue to exclude certain populations. This understanding needs to be a guiding principle of our work particularly when working with marginalised communities.

Acknowledging the value system of the profession itself further acknowledges the need to move beyond adaptations of current practices toward innovation and more radical change if we are to adequately serve marginalised populations. The majority of the papers advocated for epistemological/ideological shifts in practice [17, 18, 42] which is in line with more radical change in practice.

### Implications for research and practice

This scoping review documented the emerging practices in relation to equity. It is showing the initial movement and growing interest of the professions toward developing more equitable practices particularly for marginalised populations. While health equity is an emerging concept in the professions, it is important for us to think about the key drivers which will shift practices. Based on the scoping review, we have identified three key principles that can begin to help us shape our conceptual understanding of emerging practices which seek to address health equity: (1) partnerships with communities; (2) Global South influence; and (3) epistemological/ideological shifts.

Firstly, innovations in practice should be developed with and through marginalised communities as co-creators of practice. It is not sufficient to include marginalised groups as informants. They should form a critical part in engaging with change. Change should be driven by the needs of the community. Secondly, there needs to be greater influence of the Global South in driving emerging practices. We need to resist using decontextualised knowledge and practice from the Global North and replicating it in the Global South as a solution to local issues. Similarly, the development of emerging practices needs to have equitable participation from the Global South. Finally, in terms of the epistemological/ideological shift, the profession needs to engage with critical theory and decolonial theory and practices as it acknowledges the historical, political and social factors that shape everyday communication. This is particularly important for understanding marginalisation which was developed and sustained through colonisation and coloniality. A lack of contextualisation of marginalisation through the lens of coloniality masks the injustices as a normal part of society. The danger of a superficial approach is that it will not address the root causes of systemic injustice. In particular, we emphasise the importance of using decoloniality as a guiding frame to unmask the influence on coloniality on our society and those who continue to be marginalised and to illuminate the value system of our profession and its practices. It is with this grounding that emerging practices which address equity should be developed.

### Study limitations

Due to the complex nature of equity and the ongoing debates around its definition [5], developing a clear conceptualisation of equity for the review was challenging. This was evident in the need to continuously refine our definition throughout the review process. In particular, as a focus on equity is an emerging concept in A/SLT, developing an understanding and conceptualisation of equity through the lens of communication was a necessary process.

The review largely considered published literature which was accessible online. We acknowledge that emerging practices in the Global South may not have been formally documented and that additional further research into emerging practice in the Global South is warranted.

## Conclusion

The review found that the A/SLT profession, with its focus on communication, is working toward advancing equity through engaging with marginalised communities. The deeper analysis showed that while these movements in practice are positive, a decolonial lens is a valuable tool in addressing the systemic processes which constrain health equity. In other words, there is a need to reposition our systems and practices as framed from a colonial perspective which will provide impetus for addressing health equity. For the community of practitioners working on health equity, there is a need to consider health in a comprehensive manner, that is all social categories that impact and influence health. We argue that health equity can only be achieved when all aspects of health, and particularly communication, are considered equitably.

## Supplementary Information

**Additional file 1. **Literature review search. Example of how the literature review search was conducted for one of the databases.

**Additional file 2. **Data extraction tool. Table describing the nature of data extraction linked to the objectives of the study.

**Additional file 3. **List of included papers. Reference list for the included studies in the scoping review.

**Additional file 4. **Description of excluded papers. Table summarising the reasons for exlusion of papers from the study.

## Data Availability

Not applicable.

## Abbreviations

A/SLT: Audiology and speech-language therapy
JBI: Joanna Briggs Institute
PRISMA-SCR: Meta-Analysis Extension for Scoping Reviews
SE: Socio-economic
PWCD: People with communication disability
USA: United States of America
UK: United Kingdom
CALD: Culturally and linguistically diverse
