# Correction to: Emerging professional practices focusing on reducing inequity in speech-language therapy and audiology: a scoping review

**DOI:** 10.1186/s12939-023-01892-9

**Published:** 2023-05-24

**Authors:** Kristen Abrahams, Rizwana Mallick, Ameer S-J Hohlfeld, Thiani Pillay, Tamzyn Suliaman, Harsha Kathard

**Affiliations:** 1 Division of Communication Sciences and Disorders, Department of Health and Rehabilitation Sciences, University of Cape Town, F45 Old Main Building, Groote Schuur Hospital, Observatory, Cape Town, South Africa; 2 South African Medical Research Council, Cochrane South Africa, Francie Van Zijl Drive, Parowvallei, Tygerberg, PO Box 19070, Cape Town, 7505 South Africa; 3 Discipline of Speech Language Pathology, College of Health Sciences, University of Kwa-Zulu Natal, KwaZulu Natal, University Road, Westville, Private Bag X 54001, Durban, 4000 South Africa; 4 Chancellor Oppenheimer Library, University of Cape Town, UCT Libraries, North Lane, Rondebosch, Cape Town, 7701 South Africa; 5 Inclusive Practices Africa Research Unit, University of Cape Town, Private Bag X3, Rondebosch, Cape Town, 7701 South Africa

**International Journal for Equity in Health (2023) 22:43**

10.1186/s12939-022-01815-0

After publication of this article [1], the authors reported that the author name ‘Suliaman’ was incorrectly written as ‘Sulaiman’.

The original article [1] has been corrected.

## References

[1] Abrahams K, Mallick R, Hohlfeld AS-J (2023). Emerging professional practices focusing on reducing inequity in speech-language therapy and audiology: a scoping review. Int J Equity Health.

