# Supplementary material for: Emerging professional practices focusing on reducing inequity in speech-language therapy and audiology: a scoping review
Source: Int J Equity Health. 2023 Mar 10;22:43. doi: 10.1186/s12939-022-01815-0 (PMC10007798; doi:10.1186/s12939-022-01815-0)
Supplement: Supplementary file 1 — Additional file 1. Literature review search. Example of how the literature review search was conducted for one of the databases. [file 12939_2022_1815_MOESM1_ESM.docx]

**Additional file 1**

***Literature search strategy***

S1 – "speech therapy"

S2 – "speech therapist"

S3 – "speech pathology"

S4 – "speech pathologist"

S5 – "speech language therapy"

S7 – "speech language therapist"

S8 – "speech language pathology"

S9 – "speech language pathologist" OR

S10 – audiology

S11 – audiologist

S12 – S1 OR S2 OR S3 OR S4 OR S5 OR S6 OR S7 OR S8 OR S9 OR S10 OR S11

S13 – equit*

S14 – inequit*

S15 – marginal*

S16 – underserved

S17 – "global health"

S18 – minority

S19 – "human rights"

S20 – "global engagement"

S21 – S13 OR S14 OR S15 OR S16 OR S17 OR S18 OR S19 OR S20

S22 – "clinical practice"

S23 – practice

S24 – “service delivery”

S25 – "professional practice"

S26 – S22 OR S23 OR S24 OR S25

S27 – S12 AND S21 AND S26
