# Supplementary material for: Emerging professional practices focusing on reducing inequity in speech-language therapy and audiology: a scoping review
Source: Int J Equity Health. 2023 Mar 10;22:43. doi: 10.1186/s12939-022-01815-0 (PMC10007798; doi:10.1186/s12939-022-01815-0)
Supplement: Supplementary file 2 — Additional file 2. Data extraction tool. Table describing the nature of data extraction linked to the objectives of the study. [file 12939_2022_1815_MOESM2_ESM.docx]

**Additional file 2**

***Data extraction tool***

| **Citation** |  |
| --- | --- |
| **Reviewers** |  |

| **Objective 1** | **Level 1** analysis – documenting/describing | **Level 2** analysis – applying decolonial framework |
| --- | --- | --- |
| To synthesise the ways in which *equity is defined* in the professions | *Focus will be on understanding what lens they use to think about equity and the intersections of social categories* | *Understanding papers through colonial matrix of power – gender, race, class, language, culture, economic, sexuality, religion, etc.* |
|  |  |  |
| **Objective 2** | **Level 1** analysis – documenting/describing | **Level 2** analysis – applying decolonial framework |
| Identify and describe *innovations in practices* | *Focus will be on the level of activity – documenting key stakeholders, methodology,* | *Focus here will be on unpacking the innovation in practices/ critique* |
|  |  |  |
| **Objective 3** | **Level 1** analysis – documenting/describing | **Level 2** analysis – applying decolonial framework |
| understand and describe the *drivers for change* in clinical practice | *Documenting argument for need to explore new practices* | *Decoloniality as framing for analysis* |
|  |  |  |
