# Supplementary material for: Emerging professional practices focusing on reducing inequity in speech-language therapy and audiology: a scoping review
Source: Int J Equity Health. 2023 Mar 10;22:43. doi: 10.1186/s12939-022-01815-0 (PMC10007798; doi:10.1186/s12939-022-01815-0)
Supplement: Supplementary file 3 — Additional file 3. List of included papers. Reference list for the included studies in the scoping review. [file 12939_2022_1815_MOESM3_ESM.docx]

**Additional file 3**

***List of the included papers***

Abrahams K, Kathard H, Harty M, Pillay M. Inequity and the professionalisation of speech-language pathology. Professions and Professionalism. 2019;9(3); doi: <https://doi.org/10.7577/pp.3285>.

Bondurant LM. Breaking Down Silos: Interprofessional Collaborative Practice in Humanitarian Audiology. Seminars in Hearing. 2020;41(2):92-9; doi: [10.1055/s-0040-1708506](https://doi.org/10.1055%2Fs-0040-1708506).

Brewer KM. Clinical insights from research with New Zealand Māori. Journal of Clinical Practice in Speech-Language Pathology. 2017;19(1):9-13.

Carroll C, Guinan N, Kinneen L, Mulheir D, Loughnane H, Joyce O, et al. Social participation for people with communication disability in coffee shops and restaurants is a human right. International Journal of Speech-Language Pathology. 2018;20(1):59-62; doi: 10.1080/17549507.2018.1397748.

Cheng L-RL. Knowledge transfer between minority and majority world settings and its application to the World Report on Disability. International Journal of Speech-Language Pathology. 2013;15(1):65-8; doi: [10.3109/17549507.2012.729862](https://doi.org/10.3109/17549507.2012.729862).

Davidson B, Hill AE, Nelson A. Responding to the World Report on Disability in Australia: Lessons from collaboration in an urban Aboriginal and Torres Strait Islander school. International Journal of Speech-Language Pathology. 2013;15(1):69-74; doi: [10.3109/17549507.2012.732116](https://doi.org/10.3109/17549507.2012.732116).

Dressel A, Mkandawire-Valhmu L, Dietrich A, Chirwa E, Mgawadere F, Kambalametore S, et al. Local to global: Working together to meet the needs of vulnerable communities. Journal of Interprofessional Care. 2017;31(5):667-9; doi: [10.1080/13561820.2017.1329717](https://doi.org/10.1080/13561820.2017.1329717).

Fuller A. Speech and language therapy in Sure Start Local Programmes: a survey-based analysis of practice and innovation. International Journal of Language & Communication Disorders. 2010;45(2):182-203; doi: [10.3109/13682820902836286](https://doi.org/10.3109/13682820902836286).

Grech H, Cheng LRL. Conceptual Framework for Speech Language Pathologists to Work with Migrants: A Focus on Malta. Journal of Educational Issues. 2016;2(2):141-63; doi:10.5296/jei.v2i2.9962.

Hopf SC. Communication Capacity Research in the Majority World: Supporting the human right to communication specialist services. International journal of speech-language pathology. 2018;20(1):84-8; doi: <https://doi.org/10.1080/17549507.2018.1400101>.

Hyter YD. A Conceptual Framework for Responsive Global Engagement in Communication Sciences and Disorders. Topics in Language Disorders. 2014;34(2):103-20; doi: 10.1097/TLD.0000000000000015.

Kathard H, Pillay M. Promoting change through political consciousness: A South African speech-language pathology response to the World Report on Disability. International Journal of Speech-Language Pathology. 2013;15(1):84-9; doi: [10.3109/17549507.2012.757803](https://doi.org/10.3109/17549507.2012.757803).

Merritt B. Toward More Inclusive Gender Identity Measurement in Speech, Language, and Hearing Practice and Research. Perspectives of the ASHA Special Interest Groups. 2020;5(6):1710-5; doi: <https://doi.org/10.1044/2020_PERSP-20-00142>.

Pascoe M, Klop D, Mdlalo T, Ndhambi M. Beyond lip service: Towards human rights-driven guidelines for South African speech-language pathologists. International journal of speech-language pathology. 2018;20(1):67-74.

Penn C. Asking New Questions and Seeking New Answers. Topics in Language Disorders. 2014;34(2):168-81; doi:[10.1097/TLD.0000000000000012](http://dx.doi.org/10.1097/TLD.0000000000000012).

Penn C, Armstrong E, Brewer K, Purves B, McAllister M, Hersh D, et al. Decolonizing Speech-Language Pathology Practice in Acquired Neurogenic Disorders. Perspectives of the ASHA Special Interest Groups. 2017;2(2):91-9; doi:[10.1044/persp2.SIG2.91](http://dx.doi.org/10.1044/persp2.SIG2.91).

Pillay M, Kathard H. Renewing Our Cultural Borderlands. Topics in Language Disorders. 2018;38(2):143-60; doi: 10.1097/TLD.0000000000000151.

Pillay M, Kathard H, Samuel MA. The Curriculum of Practice: A conceptual framework for speech-language therapy and audiology practice with a black African first language clientele. South African Journal of Communication Disorders. 1997;44:109-17.

Suen JJ, Marrone N, Han H-R, Lin FR, Nieman CL. Translating Public Health Practices: Community-Based Approaches for Addressing Hearing Health Care Disparities. Semin Hear. 2019;40(01):037-48; doi: [10.1055/s-0038-1676782](https://doi.org/10.1055/s-0038-1676782).

Westby C. Implementing recommendations of the World Report on Disability for indigenous populations. International Journal of Speech-Language Pathology. 2013;15(1):96-100; doi: [10.3109/17549507.2012.723749](https://doi.org/10.3109/17549507.2012.723749).
