# Supplementary material for: Emerging professional practices focusing on reducing inequity in speech-language therapy and audiology: a scoping review
Source: Int J Equity Health. 2023 Mar 10;22:43. doi: 10.1186/s12939-022-01815-0 (PMC10007798; doi:10.1186/s12939-022-01815-0)
Supplement: Supplementary file 4 — Additional file 4. Description of excluded papers. Table summarising the reasons for exlusion of papers from the study. [file 12939_2022_1815_MOESM4_ESM.docx]

**Additional file 4**

***Description of excluded papers***

| **Reason for exclusion** | **Description** | **Papers excluded** |
| --- | --- | --- |
| No focus on equity | Papers did not meet the critera for marginalisation or intersectionality. Papers may have only focued on one aspect (e.g. culture, language, location, ethnicity etc.) | 77 |
| No focus on clinical practice | Papers focused soley on education or research without a specific focus on clinical practice. | 27 |
| Not A/SLT | Papers did not explicitly mention the professions of audiology or speech-language therapy | 1 |
| No access | The authors were unable to access the papers either online or via communication with the authors. | 6 |
